# Investigating neighbourhood environmental risk factors associated with childhood acute respiratory infection symptoms in Ethiopia mixed effect and multilevel logistic regression analysis based on EDHS 2016

**DOI:** 10.3389/fpubh.2024.1391682

**Published:** 2024-08-01

**Authors:** Jember Azanaw, Fasika Weldegebriel, Asmamaw Malede

**Affiliations:** Department of Environmental and Occupational Health and Safety, Institute of Public Health, College of Medicine and Health Sciences, University of Gondar, Gondar, Ethiopia

**Keywords:** acute respiratory infection, children, neighbourhood risk factors, under five, Ethiopia

## Abstract

**Background:**

Acute respiratory infection is an infectious illness caused by acute viral or bacterial infection. According to a 2018 WHO report, exposures to indoor and ambient air environmental pollution were contributing factors to a higher risk of respiratory problems following 7 million deaths of children under five globally. Housing conditions such as wall material, roof type, kitchen location, sanitation condition, and cooking fuel type are household-level predictors of acute respiratory disease among children under five years of age.

**Method:**

This research used EDHS-2016 secondary data, which are nationally representative. The data collection period was from January 18, 2016, to June 27, 2016. Among the 16,650 total surveys, 10,006 households that had children below 5 years of age. The outcome variable for this study was acute respiratory infection symptoms. Analyses were performed using STATA Version 17.1. The data were weighted before performing analysis to reinstate the representativeness of the sample. In the bivariable analysis, a *p* value <0.2 was used to screen for multivariable. Multicollinearity was checked using the variance inflation factor. Then, a multilevel multivariable regression model was used in this study for the analysis of acute respiratory infection symptoms and possible predictor variables. Variables with a *p* value <0.05 in multivariable regression analysis were considered statistically significant predictors.

**Results:**

Most (95.00%) households commonly used solid fuel for cooking, and household main construction materials: 81.44 and 91.03% of floors and walls of households were constructed with unprocessed natural materials, respectively. The prevalence of acute respiratory infection symptoms among children under five years of age was 7.955% (7.397, 8.551%). The findings indicated that acute respiratory infection symptoms among children under five years of age were significantly linked with the age of the children, diarrhea status, residence, region, fuel type, stool disposal, wall material, and floor material.

**Conclusion:**

Interventions should target modifiable factors such as proper stool disposal of the youngest child, informing the health effects of poor housing conditions such as improving wall and floor construction material to reduce acute respiratory infection symptoms among children under five years of age.

## Background

Acute respiratory infection (ARI) is an infectious illness caused by acute viral or bacterial infection of the breathing system, which is experienced by coughing, runny nose, sneezing, muscle pain, fever, and in the worst case may lead to shortness of breath and coughing up blood (1, 2). According to a 2018 World Health Organization (WHO) report, environmental exposures were contributing factors to a higher risk of respiratory problems, with indoor and ambient air pollution accounting for 7 million deaths of children under five globally (3). These exposures accounted for 210,777 disability-adjusted life years (DALYs) in 2015, with the highest DALYs per year in 1-year-old children (4). Approximately 1.3 million children die due to ARI annually (5).

Housing conditions have a substantial role in safeguarding people’s health (6). In the twenty-first century, most of society spends approximately 90% of their time at home, and the indoor housing condition correspondingly has a substantial influence on an inhabitant’s respiratory health (7). It is obvious that developing countries such as sub-Saharan African countries are increasing in urbanization rates. Such unintended rapid urbanization can lead to many slum dwellers, inadequate infrastructure and services, improper water and sanitation systems, and deteriorating air quality (8). In such countries, housing construction materials, accessing clean fuel, chimneys installation, number of windows, and having separate kitchens are all hidden by basic needs, drought, and many other man-made problems. However, such a housing environment influences the occurrence of ARIs by creating poor indoor air quality (2).

In particular, an innocuous and healthy living atmosphere contributes to youth growth and health (9). Because, children who have significant exposure to air pollutants at home, owing to their actively developing bodies, and tend to spend more time indoors. However, environmental factors continue to be a key risk factor that disturbs children’s health, which causes various short-and long-term impairments in children (10). Even if the problem is worldwide, environmental factors act as major risks to children’s respiratory disease among middle-and low-income countries (11).

The above issues indicated that housing conditions such as wall material, roof type, kitchen location, sanitation condition, and cooking fuel type are household-level predictors of acute respiratory disease among children under five years of age (12). Diseases of environmental origin in children can be prevented with the help of a combination of research to notice the environmental causes of disease together with science-based support (13).

Although the main studies are conducted internationally, there is still limited research on the impact of housing risk factors in the living environment on respiratory diseases among lower-developed countries. On the other hand, some include only one or a few environmental factors associated with acute respiratory symptoms (14, 15). At the same time, studies might also include limited study subjects and very specific study areas (16, 17), which are unable to show nationally representative evidence. Investigating the factors linked with acute respiratory symptoms, especially modifiable environmental risk factors, can decrease the morbidity of children due to acute respiratory disease.

Therefore, the aim of this study is to identify household-related environmental conditions affecting acute respiratory symptoms among children under five years of age in Ethiopia using the EDHS-2016 dataset. The study can provide substantial evidence to policymakers, health professionals, and other concerned stakeholders who are engaged in improving children’s health through enhanced housing conditions.

## Methods

### Data sources and sampling technique

**Figure 1:**
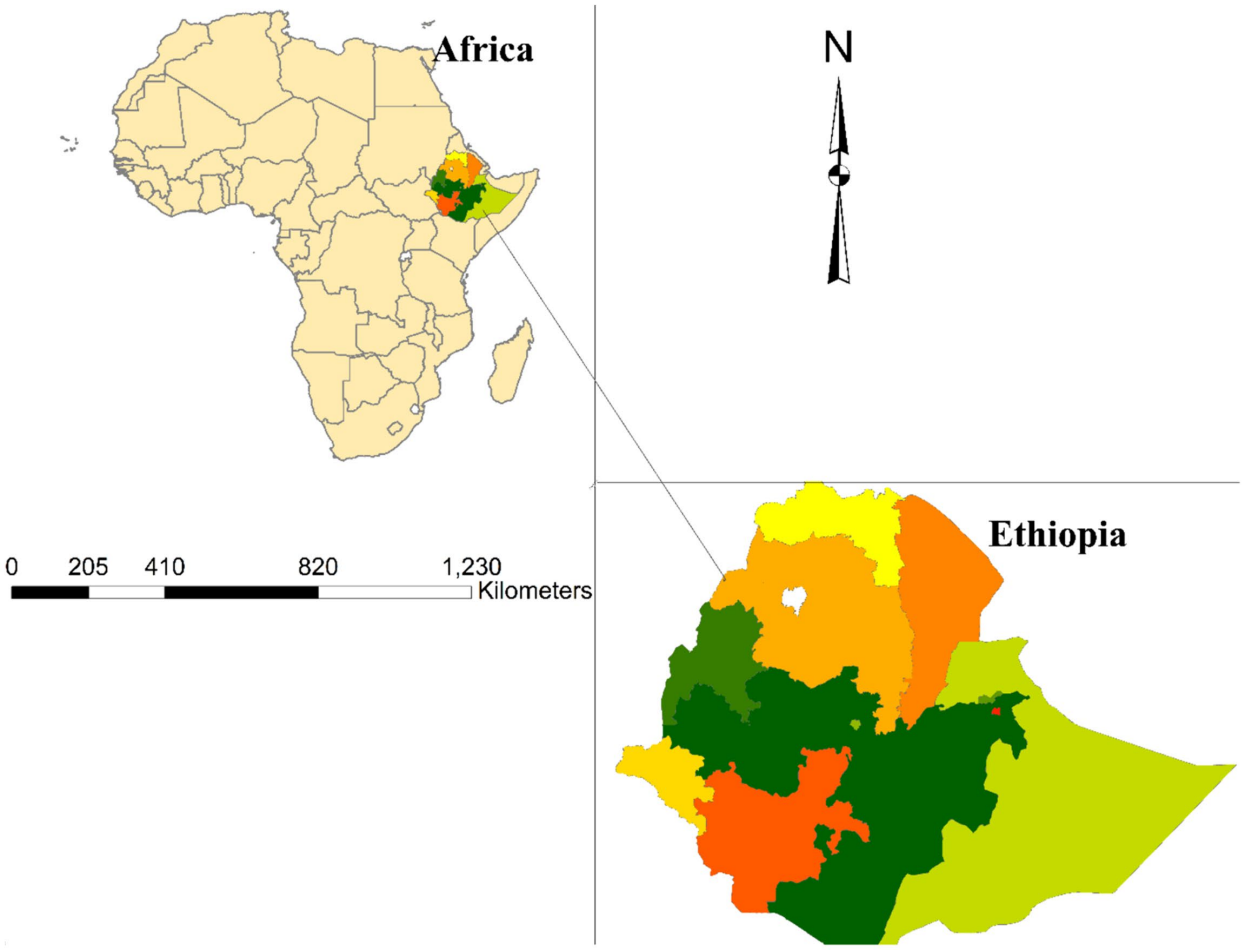
Map of Ethiopia with nine regions.

Ethiopia is the second largest population in Africa, and the country is under sub-Saharan countries. The country is divided into nine regions and two administrative cities (Figure 1).

This research used secondary data extracted from the Ethiopian Demographic and Health Survey (EDHS), 2016, a nationally representative dataset. The Ethiopian Public Health Institute (EPHI), in partnership with the Central Statistical Agency (CSA) and the Federal Ministry of Health (FMoH), employed the 2016 EDHS. The data collection period was from January 18, 2016, to June 27, 2016. The sample frame used in the survey EDHS, 2016, was stratified into urban and rural domains and further into regions and districts to obtain an adequate representation of each strata. Ethiopian Population and Housing Census sampling frame conducted in 2007 through probability proportional to the unit size. Approximately 16,650 primary sampling units, including 11,418 rural and 5,232 urban units, were selected among the households. Among the selected households, 10,006 had children below 5 years of age and were included in this study.

### Variables of the study

#### Outcome variable

The outcome variable for this study was acute respiratory symptoms. Children with a cough in the last 2 weeks, faced problems in the chest, or a blocked or running nose, and short, rapid breaths were recorded as having acute respiratory symptoms (18). The questionnaire asked about the cough in the last 2 weeks was represented 0 for “no” and 1 for “yes”; short and rapid breaths were transformed into 0 for “no” and 1 for “yes.” Then, the sum was represented as 0 for free of any symptom, 1 for one of them (cough in the last 2 weeks or short and rapid breaths), or for the combination of cough in the last 2 weeks and short, rapid breaths, which is the target variable.

#### Predictor variables

##### Cooking fuel type

Coal/charcoal, wood, agricultural residue/shrubs, animal dung, and kerosene were grouped under solid fuels, whereas liquefied petroleum gas (LPG), electricity, and biogas were classified as clean fuels.

##### Sanitation facilities

Households with pit latrines without a slab or platform, hanging latrines or bucket latrines, and open defecation were categorized as having unimproved sanitation. The remaining households with flush/pour flush to piped sewer systems, septic tanks, or pit latrines; ventilated improved pit latrines, composting toilets, or pit latrines with slabs were improved in sanitation facilities. For analysis, unimproved and improved sanitation facilities were represented by 0 and 1, respectively.

##### Housing construction materials

The main housing materials were natural and finished housing materials. Based on these main roof materials constructed categorized as natural roofing with no roof, thatch/mud, sod, rustic mat/plastic sheet, reed/bamboo, wood planks, and cardboard (19) were denoted by 0, and the remaining materials, including metal/corrugated iron, wood, calamine/cement fibre/asbestos, ceramic tiles, cement, and roofing shingles, were classified as finished roofing, represented by 1. On the other hand, floors categorized as natural floors, which include earth/sand, wood planks, and palm/bamboo (20), were denoted as 0, and houses constructed from parquet or polished wood, vinyl or asphalt strips/plastic tile, ceramic tiles, cement, and carpet were categorized as finished floors, denoted as 1. Houses constructed using natural wall materials such as no walls, cane/palm/trunks/bamboo/ree, dirt, bamboo with mud, stone with mud, uncovered adobe, plywood, cardboard, and reused wood were coded as 0, and house walls constructed with cement, stone with lime/cement, bricks, cement blocks, covered adobe, and wood planks/shingles (21) were recorded as finished walls and denoted by 1.

##### Diarrhea disease

Study subjects were asked whether the children had experienced diarrhea in the last 2 weeks in the form of yes, no, and do not know. Then, “yes” was recorded as 1, and “no” was represented by 0.

##### Disposal of youngest child’s stools when not using the toilet

Used toilet/latrine, put/rinsed in toilet/latrine, buried were recoded as proper (1) whereas put/rinsed into drain or ditch, throw into garbage, left in the open/not disposed of, and other represented as improper (0).

### Data analysis

Descriptive and analytical analyses were performed to show the findings of the study. The data underwent a meticulous weighting process prior to any data analysis to restore and uphold the sample’s representativeness. This crucial step ensured that each data point contributed appropriately according to predetermined demographic or statistical criteria, thereby correcting any potential biases and allowing for accurate and reliable analyses. Bivariable logistic regression analysis was used to screen the association between acute respiratory symptoms and the independent variables or risk factors separately by forward stepwise variable selection. Due to the hierarchical structure of demographic health survey data, where individual responses are nested within households, and households are further nested within communities or regions, a multilevel logistic regression analysis was conducted. This analytical approach allows for the accommodation of the nested data structure, accounting for the potential correlations within clusters at different levels. By doing so, it enables the estimation of more accurate standard errors and the partitioning of variance at each level, providing a deeper understanding of the factors influencing ARISs across different hierarchical levels.” The dependent variables included in the analysis were selected using the forward selection method. Then, variables in the bivariable with a *p* value <0.2 were included in the multivariable logistic regression model. Variables in the multivariable logistic regression model with a *p* value <0.05 were considered significant predictors. Four models: Null model with only the target variable, model-I consisting of household and individual characteristics, model-II including community-level factors, and model-III encompassing both individual-and community-level characteristics were fitted. Multicollinearity was checked for the second, third and final models using the variance inflation factor (VIF). To examine the discriminatory performance of this logistic regression model, receiver operating characteristic (ROC) analysis (22) was conducted. In addition, a goodness of fit test was performed using information criteria (AIC, BIC, and DIC). All statistical analyses were performed using STATA Version 17.1.

## Results

### Sociodemographic characteristics

The table represents some of the sociodemographic characteristics of the study subjects. The results show that most of the mothers of the children were married (93.06%), and slightly more than half (51.53%) of the children were males. A total of 8,667 (81.45%) of the respondents resided in rural areas. The majority (64.26%) of the mothers had no education, and only a few (3.67%) of them had completed higher education (Table 1).

**Table 1:** Frequency and percentage of respondents’ sociodemographic characteristics, EDHS 2016 (*N* = 10,006).

Attribute	Categories	Frequency (%)
Age of child in a month	<13	2,663 (25.03)
	13–29	2,765 (25.98)
	30–44	2,554 (24.00)
	45–59	2,659 (24.99)
Marital status of mothers	Married	9,903 (93.06)
	Unmarried	738 (6.94)
Number of U5C	1	3,759 (35.33)
	2	4,518 (42.46)
	≥3	2,014 (18.93)
Sex of U5C	Male	5,483 (51.53)
	Female	5,158 (48.47)
Mothers educational status	No education	6,838 (64.26)
	Incomplete primary	2,444 (22.97)
	Complete primary	234 (2.20)
	Incomplete secondary	633 (5.95)
	Complete secondary	101 (0.95)
	Higher	391 (3.67)
Husband/partner’s educational level	No education	4,928 (49.24)
	Primary	3,220 (32.17)
	Secondary	1,015 (10.14)
	Higher	770 (7.69)
	Do not know	75 (0.75)
Residence	Rural	8,667 (81.45)
	Urban	1,974 (18.55)
Region	Tigray	1,033 (9.71)
	Afar	1,062 (9.98)
	Amhara	977 (9.18)
	Oromia	1,581 (14.86)
	Somali	1,505 (14.14)
	Benishangul	879 (8.26)
	SNNPR	1,277 (12.00)
	Gambella	714 (6.71)
	Harari	605 (5.69)
	Addis Ababa	461 (4.33)
	Dire Dawa	547 (5.14)

### Neighbourhood environmental factors

Table 2 shows different types of household environmental conditions from the EDHS-2016 data, which are linked directly or indirectly to children’s health, including acute respiratory symptoms. Solid fuel categories were commonly (95.00%) used by households and the households’ main construction materials: floor and walls (81.44 and 91.03%, respectively) were constructed with unprocessed natural materials. Nearly one-tenth (10.89%) of the children experienced diarrhea (Table 2).

**Table 2:** Household environmental conditions linked with acute respiratory symptoms in Ethiopia based on EDHS 2016.

Attribute	Categories	Frequency (%)
Main floor material	Natural	8,666 (81.44)
	Finished	1,975 (18.56)
Main wall material	Natural	9,686 (91.03)
	Finished	955 (8.97)
Main roof material	Natural	5,513 (51.81)
	Finished	5,128 (48.19)
Type of sanitation facility	Unimproved	8,915 (83.78)
	Improved	1,726 (16.22)
Fuel type	Solid	10,109 (95.00)
	Clean	532 (5.00)
Disposal of youngest child’s stools	Improper	8,341 (78.39)
	proper	2,300 (21.61)
Time to get water	On-premises	1,789 (16.81)
	≤30	4,986 (46.86)
	>30	3,866 (36.33)
Diarrhea in the past 2 weeks	No	8,916 (89.11)
	Yes	1,090 (10.89)

### Prevalence and factors associated with acute respiratory symptoms

**Figure 2:**
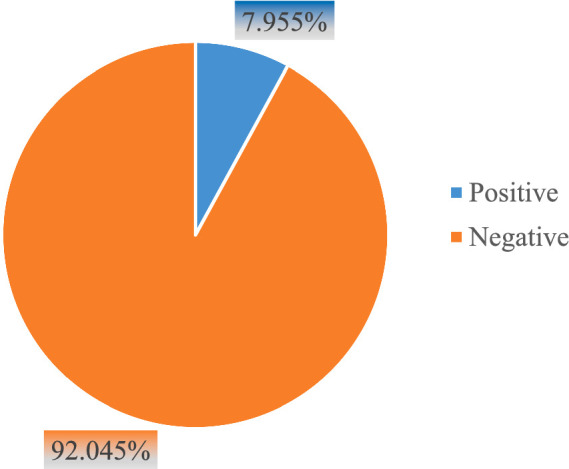
Prevalence of acute respiratory infection symptoms among children under five years of age in Ethiopia, EDHS 2016.

Based on the EDHS 2016 dataset, the prevalence of acute respiratory symptoms among children under five years of age was 7.955% (7.397, 8.551%) (Figure 2).

### Measures of random effect and model fit statistics

The ICC value of the null model in the table indicated that 25% of the variation in ARI symptoms was due to the clustering effect. The PCV value result of model II revealed that 33% of the difference in ARI symptoms among children was explained by community-level factors. On the other hand, the MOR value indicated that the children in the higher risk cluster were 2.757 times more likely to experience ARI symptoms than the children in the lower risk cluster. The final model showing the smallest values of AIC, BIC and DIC was considered the best fit model (Table 3).

**Table 3:** Measures of variation and model fitness for both improved water sources and sanitation in Ethiopia.

Parameters	Empty model 0	Model I	Model II	Model III
Measures of variations				
Variance	1.149	1.068	0.713	0.665
PCV (%)	Reference	7.050	33.240	7.218
ICC (%)	25.890	24.512	17.816	16.818
MOR	2.757	2.669	2.230	2.170
Model fitness test statistics				
AIC	5,284.448	5,216.711	5,174.793	**4,899.178**
BIC	5,298.869	5,296.032	5,268.536	**5,050.608**
DIC	5,289.544	5,216.156	5,148.7934	**4,857.178**

AIC, Akaike’s information criterion; BIC, Bayesian’s information criterion; DIC, Deviance information criterion; ICC, intraclass correlation coefficient; PCV, variance partition coefficients. Bold values means Fitted with lowest AIC, BIC and DIC values.

### Sensitivity and specificity test of the model

**Figure 3:**
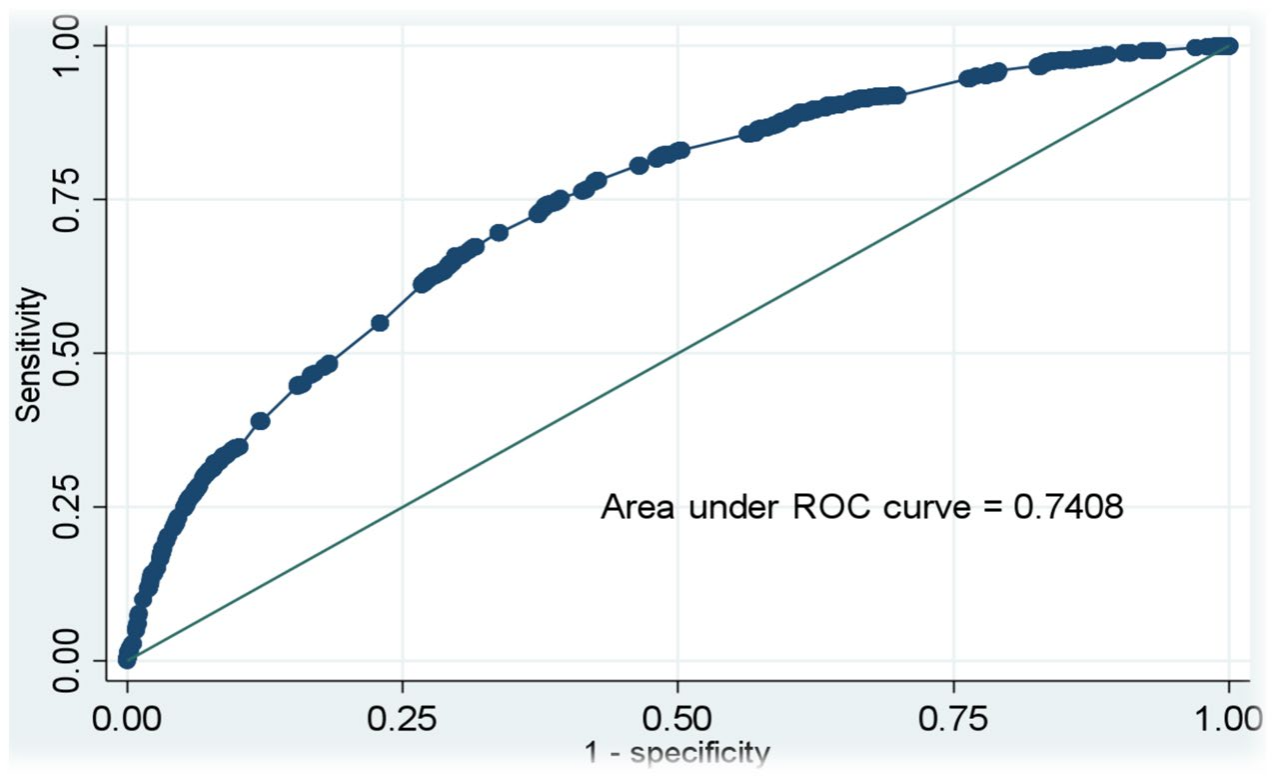
Receiver operating characteristic curves representing the diagnostic accuracy of the final logistic regression model for ARI symptoms are shown in Table 4.

Figure 3 presents the sensitivity and specificity or discriminant power of the ARI symptoms model among children under five years of age. We found that the model-predicted probability of experiencing ARI symptoms was 74% success. This indicated that the models were acceptable in discriminatory power (Figure 3).

### Multilevel multivariable binary regression of factors associated with acute respiratory symptoms

The findings in the factor analysis in Table 4 indicated that acute respiratory symptoms among children under five years of age were significantly linked with the age of the children, diarrhea status, residence, fuel type, stool disposal, wall material, and floor material.

**Table 4:** Factor analysis of acute respiratory symptoms using multilevel multivariable regression.

	Null model	Model I	Model II	Model III
Attributes				AOR (95%CI)
**Age of children in months**				
<13		1.76 (1.42, 2.18)**		1.73 (1.40, 2.14)**
13–29		1.58 (1.28, 1.96)**		1.60 (1.29, 1.98)**
30–44		1.17 (0.93, 1.48)		1.16 (0.92, 1.47)
45–59		1.00		1.00
**Number of children**				
1		1.00		1.00
2		1.50 (1.21, 1.86)*		1.37 (0.74, 2.54)
≥3		1.20 (0.97, 1.49)		1.16 (0.61, 2.18)
**Fuel type**				
Solid		7.12 (4.55, 1.119)**		1.74 (1.28, 2.38)**
Clean		1.00		1.00
**Stool disposal**				
Improper		1.03 (0.85, 1.24)		0.84 (0.71, 0.99)*
Proper		1.00		1.00
**Roof material**				
Natural		1.22 (1.05, 1.43)*		1.15 (0.96, 1.38)
Finished		1.00		1
**Wall material**				
Natural		1.25 (1.12, 1.75)*		2.10 (1.56, 2.84)**
Finished		1.00		1.00
**Floor material**				
Natural		1.22 (1.05, 1.43)*		4.30 (3.19, 5.79)**
Finished		1.00		1.00
**Sanitation facilities**				
Unimproved		0.84 (0.64, 1.11)		0.92 (0.72, 1.17)
Improved		1.00		1.00
**Diarrhea status**				
Negative		1.00		1.00
Positive		4.75 (4.02, 5.61)**		3.95 (3.31, 4.71)**
**Toilet status**				
Shared		1.10 (0.88, 1.38)		1.00 (0.85, 1.18)
Not shared			1.00	1.00
**Residence**				
Rural			1.82 (1.76, 1.98)*	1.55 (1.25, 1.91)**
Urban			1.00	1.00
**Regions**				
Tigray			2.86 (1.52, 5.41)*	2.27 (1.14, 4.50)*
Afar			1.19 (0.60, 2.36)	0.88 (0.42, 1.83)
Amhara			1.70 (0.88, 3.29)	1.30 (0.64, 2.67)
Oromia			2.50 (1.32, 4.76)*	2.03 (1.02, 4.07)*
Somalia			0.57 (0.29, 1.13)	0.46 (0.22, 0.96)*
Benishangul			0.31 (0.14, 0.71)*	0.24 (0.10, 0.57)*
SNNPR			1.44 (0.75, 2.78)	1.03 (0.50, 2.10)
Gambella			0.66 (0.32, 1.38)	0.47 (0.21, 1.03)
Harari			0.36 (0.15, 0.83)*	0.28 (0.11, 0.66)*
Addis Ababa			1.00	1.00
Dire Dawa			1.00 (0.49, 2.04)	0.68 (0.31, 1.46)
VIF	3.67	3.48	2.35	1.33

1.00 = Reference; *, *p* value < 0.05; **, *p* value < 0.001; VIF, variance inflation factor.

These findings revealed that children younger than 13 months and 13–29 months in age had 1.73 (AOR = 1.73, 95% CI = 1.40–2.14) and 1.60 (AOR = 1.60, 95% CI = 1.29–1.98) times higher odds of experiencing acute respiratory symptoms than their 45–59-month-old children.

Children under five years of age who were associated with solid fuel use were nearly two times (OR = 1.74, 95% CI = 1.28–2.38) more likely to have acute respiratory symptoms than their clean fuel counterparts.

The chance of experiencing acute respiratory symptoms among children under five years of age was two times higher (AOR = 2.10, 95% CI = 1.56–2.84) among those who lived in a house with natural main wall material than among those who survived in a house with finished main construction materials. On the other hand, the odds of acute respiratory symptoms among children under five years of age living in a house with natural main floor material was more than four times (AOR = 4.30, 95% CI = 3.19–5.79) that of children under five years of age living in a house with finished main floor materials.

Our findings also revealed that when the children under five years of age moved from where improper stool disposal of the youngest child took place to where proper stool disposal of the youngest child took place, acute respiratory symptoms were reduced by 16% (AOR = 0.84, 95% CI = 0.71–0.99).

The odds of acute respiratory symptoms among children under five years of age were more than four times (AOR = 3.95, 95% CI = 3.31–4.71) higher among children who experienced diarrhea than among children not affected by diarrhea.

The likelihood of acute respiratory symptoms among children under five years of age was four times higher (AOR = 3.95, 95% CI = 3.31–4.71) among children who lived in rural areas than among their children living in urban areas.

Children in the Tigray region had a 2.27 (AOR = 2.27, 95% CI = 1.14–4.50) chance of experiencing ARI symptoms, and those in the Oromia region had a 2.03 (AOR = 2.03, 95% CI = 1.02–4.07) more likely than the children found in Addis Ababa. In contrast, the children living in Somalia, Benishangul, and Harari were reduced by 54% (AOR = 0.46, 95% CI = 0.22–0.96), 76% (AOR = 0.24, 95% CI = 0.10–0.57) and 72% (AOR = 0.28, 95% CI = 0.11–0.66), respectively, compared to the children residing in Addis Ababa. The VIF value of all models disclosed the absence of a Multicollinearity effect (Table 4).

## Discussion

The aim of this study was to find neighbourhood environmental factors influencing the prevalence of acute respiratory symptoms among children under five years of age using the EDHS 2016. The study included 10,006 children younger than five years. Seven hundred and ninety-six (7.955% (7.397, 8.551%)) of the children experienced acute respiratory symptoms. The factor analysis of the findings of the study indicated that acute respiratory symptoms among children under five years of age were significantly associated with age, diarrhea status, residence, fuel type, stool disposal of youngest child, main wall material, and floor material.

This finding revealed that the prevalence of ARI symptoms was negatively linked with the age of the children. Younger children increased in experiencing acute respiratory symptoms compared to children near the age of five years. In reality, as their age increases, their immune system will be advanced to resist disease (23), they will advance in consciousness, and this is the time they get school where they are educated, including health and safety. This finding is in line with the studies performed in Bangladesh (24) and Nepal (25), which were conducted on ARI symptoms.

The main construction materials of the house (wall and floor) were strongly associated with acute respiratory symptoms among children under five years of age. The probability of experiencing ARI symptoms among children under five years of age was higher among children who resided in a house with a natural main wall and floor materials. Such poor housing standards are due to lower socioeconomic status and cultural acceptance leading to poor ventilation, supporting growth molds, home dampness (26–28), and difficulty cleaning acting as the source of dust particles limiting healthy life, including ARI symptoms among children (29–32). According to the findings of the studies performed in Lao PDR (33), main wall and floor materials were significantly associated with ARI symptoms, which is in line with this study. However, this finding contradicts the study performed in Punjab, Pakistan, in the case of wall main construction material and ARI symptom association (31). However, roof construction materials were not a statistically significant predictor of ARI symptoms in the children. This could be due to the height where the children had not come into direct contact with the roof rather than the wall and floor. This was consistent with an earlier study performed in Punjab, Pakistan (31). Therefore, housing conditions are a determinant of children’s health, including acute respiratory infections (34–37).

This study also revealed that the children under five years of age who found where improper stool disposal of the youngest child took place were more likely to have acute respiratory symptoms than the children who lived where proper stool disposal of the youngest child took place. Improper or unsafe stool disposal includes being put/rinsed into drains or ditches, being thrown into garbage, being, being left open/not disposed of, which is related to housing environmental contamination and creating bad odours, especially for children.

The probabilities of ARI symptoms among children under five years of age were increased among children who experienced diarrhea compared to children not affected by diarrhea. A possible explanation for this finding is that diarrhea, especially acute diarrhea, might lead to the loss of micronutrients and dryness, suppress the immune system (38), and expose children to bad smells, which are associated with respiratory problems. The study confirms previous findings in Ghana and Brazil (38) and Pakistan (18).

This study found that the likelihood of acute respiratory symptoms among children under five years of age was higher among children who lived in rural areas than among children who lived in urban areas. People who live in urban areas have a higher chance of being educated, wealthy, and exposed to information on disease prevention, including respiratory problems and awards for childcare, and they are near health facilities as well or in general. The standard of living is better in urban areas. These and other reasons might cause such differences in acute respiratory symptoms among children. This finding was consistent with other past studies (39–41) in different parts of the world.

The other factor statistically associated with acute respiratory symptoms other than the children was the type of fuel used for cooking, holding other factors constant. Children under five years of age who were connected to solid fuel use were more likely to develop acute respiratory symptoms than children who used clean fuel for cooking. Solid fuels used for cooking are sources of different gases, such as CO2, NO2, CO, methylene chloride, dioxins and respirable particulate matter (PM2.5) (42–45), which are the most health-harmful indoor air pollutants (46) where children spend more of their time. This idea is consistent with previous studies revealing the impact of solid fuel use on childhood respiratory problems (12, 39, 47). Children lived in Tigray and in Oromia; regions were affected by ARI symptoms compared with the children found in Addis Ababa. The reason behind this difference could be that Addis Ababa is the capital city where most of the economy, health and other related infrastructures fulfilled and satisfied community needs. These economy, health and other related infrastructures also enable childcare, including preventing acute respiratory infections. On the other hand, children living in the Somalia, Benishangul, and Harari regions were lower in experiencing ARI symptoms than children residing in Addis Ababa. A possible explanation for this variation is that during data collection, more vulnerable children for acute respiratory infections might not be found since there are desert areas that are difficult to access. However, the children lived in towns and cities where good health and safety take place in the regions could have a higher chance of being part of the survey.

### Limitation

The cross-sectional design limits causal inferences, and reliance on self-reported data may introduce biases. One significant limitation of the study is the highly skewed distribution of responses within most explanatory variables. Such disproportionate distribution between categories could influences the reliability and interpretability of regression outputs.

## Conclusion

Based on factor analysis findings, acute respiratory infection symptoms among children under five years of age were significantly associated with age of child, diarrhea status, residence, region, fuel type, stool disposal of youngest child, main wall material, and floor material. Interventions should be targeted in reducing acute respiratory infection symptoms among children under five years of age through proper stool disposal of the youngest child, informing the health effects of poor housing conditions such as improving wall and floor construction material. Hence, NGOs conserving under five children and the regional and federal governments of Ethiopia should focus on improving living/housing conditions and fuel types used for cooking and reducing diarrheal disease to reduce the effect of acute respiratory problems in children under five years of age.

## Data availability statement

This study utilized secondary data from publicly available datasets, which can be accessed at (48).

## Author contributions

JA: Conceptualization, Data curation, Formal analysis, Investigation, Methodology, Software, Supervision, Validation, Writing – original draft, Writing – review & editing. FW: Investigation, Visualization, Writing – review & editing. AM: Conceptualization, Investigation, Methodology, Software, Supervision, Validation, Writing – review & editing.
