# Supplementary material for: Investigating neighbourhood environmental risk factors associated with childhood acute respiratory infection symptoms in Ethiopia mixed effect and multilevel logistic regression analysis based on EDHS 2016
Source: Front Public Health. 2024 Aug 1;12:1391682. doi: 10.3389/fpubh.2024.1391682 (PMC11328150; doi:10.3389/fpubh.2024.1391682)
Supplement: Supplementary file 1 [file Table_1.DOCX]

|  | Diar rh~s | floo r_~l | wall  _m~ l | roof  _m~ l | stoo l_~l | fuel  _t~e | sani ta~s | mar rit~s | num ber~ n | ageo f_~n | time to~r | Matern al_e~n | Hasban d_ed~n |
| --- | --- | --- | --- | --- | --- | --- | --- | --- | --- | --- | --- | --- | --- |
| Diarrhe a_s~s | 1 |  |  |  |  |  |  |  |  |  |  |  |  |
| floor_m | - | 1 |  |  |  |  |  |  |  |  |  |  |  |
| ate~l | 0.00 |  |  |  |  |  |  |  |  |  |  |  |  |
|  | 61 |  |  |  |  |  |  |  |  |  |  |  |  |
| wall_m | - | 0.49 | 1 |  |  |  |  |  |  |  |  |  |  |
| ater~l | 0.01 | 72 |  |  |  |  |  |  |  |  |  |  |  |
|  | 92 |  |  |  |  |  |  |  |  |  |  |  |  |
| roof_m | 0.00 | 0.35 | 0.25 | 1 |  |  |  |  |  |  |  |  |  |
| ater~l | 34 | 04 | 21 |  |  |  |  |  |  |  |  |  |  |
| stool_di | 0.04 | 0.15 | 0.11 | 0.11 | 1 |  |  |  |  |  |  |  |  |
| sp~l | 21 | 87 | 33 | 74 |  |  |  |  |  |  |  |  |  |
| fuel_ty | - | 0.41 | 0.33 | 0.21 | 0.05 | 1 |  |  |  |  |  |  |  |
| pe | 0.01 | 52 | 46 | 58 |  |  |  |  |  |  |  |  |  |
|  | 87 |  |  |  |  |  |  |  |  |  |  |  |  |
| sanitati | - | 0.54 | 0.42 | 0.29 | 0.15 | 0.40 | 1 |  |  |  |  |  |  |
| on~s | 0.01 | 32 | 88 | 43 | 57 | 42 |  |  |  |  |  |  |  |
|  | 89 |  |  |  |  |  |  |  |  |  |  |  |  |
| marrital | 0.02 | - | 0.00 | - | - | - | - | 1 |  |  |  |  |  |
| _s~s | 19 | 0.01 | 97 | 0.01 | 0.00 | 0.00 | 0.00 |  |  |  |  |  |  |
|  |  | 17 |  | 82 | 81 | 11 | 43 |  |  |  |  |  |  |
| number | - | - | - | - | 0.09 | - | - | 0.04 | 1 |  |  |  |  |
| of_c~n | 0.04 | 0.15 | 0.02 | 0.09 | 59 | 0.08 | 0.05 | 18 |  |  |  |  |  |
|  | 35 | 17 | 66 | 65 |  | 57 | 41 |  |  |  |  |  |  |
| ageof_c | - | - | - | - | - | - | - | - | - | 1 |  |  |  |
| hil~n | 0.12 | 0.04 | 0.01 | 0.00 | 0.17 | 0.01 | 0.01 | 0.00 | 0.01 |  |  |  |  |
|  | 11 | 29 | 91 | 99 | 75 | 76 | 6 | 08 | 63 |  |  |  |  |
| timeto_ | 0.00 | 0.07 | 0.04 | - | - | 0.02 | 0.03 | 0.01 | 0.01 | - | 1 |  |  |
| get~r | 24 | 45 | 04 | 0.02 | 0.04 | 43 | 06 | 04 | 46 | 0.00 |  |  |  |
|  |  |  |  | 38 | 53 |  |  |  |  | 64 |  |  |  |
| Matern | 0.01 | 0.42 | 0.25 | 0.24 | 0.11 | 0.35 | 0.32 | - | - | - | - | 1 |  |
| al_e~n | 23 | 41 | 11 | 63 | 67 | 77 | 64 | 0.04 | 0.20 | 0.08 | 0.05 |  |  |
|  |  |  |  |  |  |  |  | 18 | 47 | 4 | 16 |  |  |
| Hasban | 0.03 | 0.38 | 0.21 | 0.23 | 0.13 | 0.28 | 0.30 | - | - | - | - | 0.5482 | 1 |
| d_ed~n | 1 | 6 | 83 | 32 | 5 | 57 | 24 | 0.02 | 0.14 | 0.05 | 0.08 |  |  |
|  |  |  |  |  |  |  |  | 13 | 86 | 71 | 31 |  |  |
